# From cells to antibodies: age-specific immune shifts in children during the COVID-19 pandemic

**DOI:** 10.3389/fimmu.2026.1849065

**Published:** 2026-09-03

**Authors:** Huijing Bao

**Affiliations:** Laboratory Science Department, Tianjin University Children’s Hospital/Tianjin Children’s Hospital, Tianjin, China

**Keywords:** age stratification, children, complete blood count, COVID-19, hygiene hypothesis, immunoglobulin E

## Abstract

**Background:**

The COVID-19 pandemic and associated containment measures could have influenced children’s immune system development through reduced microbial exposure and lifestyle changes. Hematological parameters and immunoglobulin E (IgE) are key indicators for immune status, but large-scale, age-stratified studies are lacking.

**Methods:**

Children under 18 years were enrolled during the first two weeks of December each year from 2017 to 2024 at Tianjin Children’s hospital. Data from 2019 and 2022 were excluded to minimize COVID-19 pandemic effects. Cases with missing age/sex or IgE values outside the linear range were removed. Complete blood count (CBC) data were obtained from emergency department patients (n=126, 617), and IgE levels from inpatient admissions patients (n=6, 055). General linear models (GLM) adjusted for sex and age were used to compare pre-pandemic (2017 and 2018) and post-pandemic groups (2023 and 2024). Effect sizes (η²) were calculated. Age-stratified analyses were performed in five subgroups: ≤1 year, 2–3 years, 4–6 years, 7–12 years, and 13–18 years. Baseline characteristics were compared between cohorts using chi-square test for sex and Mann-Whitney U test for age.

**Results:**

Baseline comparison showed no significant sex difference between the CBC and IgE cohorts (χ² = 3.111, *p* = 0.078; Fisher’s exact test *p* = 0.079), but age differed significantly (Mann-Whitney U = 3, 535, 702.5, Z = –130.805, *p* < 0.001). In the whole-age model, only basophils showed a significant difference (η²=0.038, *p* < 0.001); other CBC parameters had η²≤0.001. Age-stratified analysis revealed an inverted U-shaped trend for basophils, with the largest effect in children aged 4–6 years (η² = 0.051). For IgE, the whole-age model showed a small increase post-pandemic (η² = 0.013, *p* < 0.001). Age-stratified analysis demonstrated that the difference was confined to children aged 7–18 years (η² = 0.017–0.018, *p* < 0.01), with no significant changes in younger age groups.

**Conclusions:**

The COVID-19 pandemic was associated with modest increases in basophils and IgE in children, with distinct age-dependent patterns: basophils peaked in preschool children (4–6 years), while IgE increased only in adolescents (7–18 years). These findings support the hygiene hypothesis and suggest the age stratified pattern of Th2-skewed immune shifts across childhood. However, all effect sizes were small, indicating that routine CBC and IgE are not sensitive markers for individual monitoring.

## 1 Introduction

The coronavirus disease 2019 (COVID-19) pandemic prompted unprecedented public health interventions, including lockdowns, social distancing, and enhanced hygiene practices (1, 2). While these measures were crucial to control viral spread, they drastically reduced children’s exposure to common pathogens and environmental microbes (3, 4). According to the hygiene hypothesis, diminished microbial exposure during early life may predispose to allergic diseases by shifting the immune system toward a T-helper type 2 (Th2) phenotype (5–7).

Complete blood count (CBC) parameters, particularly basophils and eosinophils, are involved in Th2 immune responses and allergic inflammation (8). Immunoglobulin E (IgE) is a hallmark of atopy and allergic sensitization (9). Changes in these markers during the pandemic could provide insights into whether pandemic-related lifestyle changes have influenced the developing immune system in children (10–12).

Previous studies often combined all pediatric ages or used broad categories, potentially missing age-specific windows of vulnerability (13–17). Given the highly age-dependent nature of immune system maturation (18, 19), we conducted a large-scale, age-stratified analysis using data from a single tertiary hospital to evaluate changes in CBC parameters and IgE before and after the pandemic onset.

## 2 Materials and methods

### 2.1 Study population

Data were collected from children aged 0–18 years from Tianjin Children’s Hospital during the first two weeks of December from 2017 to 2024. To avoid transitional period effects, data from 2019 and 2022 were excluded, and the comparison is between 2017/2018 and 2023/2024. Although the main acute pandemic years (2019–2022) were partially excluded to minimise direct infection-related confounders, comparing the pre-pandemic (2017–2018) and post-pandemic (2023–2024) periods still permits evaluation of the broader pandemic-era impact on children’s immune markers. Cases with missing age or sex were removed. IgE were detected by Roche Cobas e801 (electrochemiluminescence immunoassay), with the linear range from 0.1–2500 IU/mL. The values outside the linear range of the assay were excluded. CBC results were obtained from emergency department patients (n=126, 617), and IgE results from inpatient admissions patients (n=6, 055). Exclusion criteria also included: (1) history of hematologic disorders, autoimmune diseases, or malignancy; (2) use of immunomodulators or systemic glucocorticoids within one month.

### 2.2 Baseline comparison between cohorts

To assess comparability between the two data sources, we compared sex distribution using the chi-square test and age using the Mann-Whitney U test (due to non-normal distribution). Baseline comparison revealed no significant sex difference (χ² = 3.111, *p* = 0.078; Fisher’s exact test *p* = 0.079), but age differed significantly (Mann-Whitney U = 3, 535, 702.5, Z = –130.805, *p* < 0.001). Therefore, in subsequent analyses, age was included as a covariate in all models to adjust for this difference.

### 2.3 Data collection

From the hospital information system, we extracted: (1) age (days for CBC and years for IgE); (2) sex; (3) CBC parameters: white blood cells (WBC), lymphocytes (LYMPH), neutrophils (NEUT), eosinophils (EOS), basophils (BASO); (4) IgE; (5) date of examination. Based on the official declaration of the pandemic (20), children examined before January 1, 2020 were classified as pre-pandemic, and those examined on or after as post-pandemic.

### 2.4 Statistical analysis

Analyses were performed using SPSS 26.0. General linear models (GLM) were constructed with each CBC parameter or IgE as dependent variable, pandemic group (pre- vs post-pandemic) and sex as fixed factors, and age as covariate. Effect sizes were reported as partial Eta-squared (η²). Age-stratified analyses were conducted in five subgroups: ≤1 year, 2–3 years, 4–6 years, 7–12 years and 13–18 years. Significance was set at α=0.05 (two-tailed). Given the large sample size, effect sizes were prioritized for interpretation. For eta-squared (η²) in ANOVA, conventional benchmarks: η² ≥ 0.01 small, ≥ 0.06 medium, ≥ 0.14 large (21).

## 3 Results

### 3.1 Participant characteristics

The CBC analysis included 126, 617 children (48, 810 pre-pandemic, 77, 807 post-pandemic). Median age was 5 years (IQR 2–7), and 55% were male. The IgE analysis included 6, 055 children (2, 360 pre-pandemic, 3, 695 post-pandemic) with a little lower sex distribution (39% male). Baseline comparison between the two cohorts showed no significant sex difference (χ² = 3.111, *p* = 0.078; Fisher’s exact test *p* = 0.079), but a significant age difference was observed (Mann-Whitney U = 3, 535, 702.5, Z = –130.805, *p* < 0.001). All data were collected in the first two weeks of December, controlling for seasonal variation.

### 3.2 Hematological parameters

#### 3.2.1 Whole-age model

In the whole-age GLM (**Table 1**), only basophils showed a significant difference between the two periods (η² = 0.038, *p* < 0.001). Although WBC, lymphocytes, neutrophils, and eosinophils were statistically significant (*p* < 0.05), their effect sizes were negligible (η² ≤ 0.001). The group×sex interaction was non-significant for all parameters. Age had the strongest effects on lymphocytes (η² = 0.083) and neutrophils (η² = 0.023).

**Table 1:** Whole-age general linear model results for CBC parameters.

Parameter η² (*p*)	Group (pre- vs post-pandemic)	Age	Gender	Gender×group
WBC	0.000 (0.024)	0.000 (0.002)	0.000 (<0.001)	0.000 (0.253)
Lymphocytes	0.001 (<0.001)	0.083 (<0.001)	0.000 (0.039)	0.000 (<0.001)
Neutrophils	0.001 (<0.001)	0.023 (<0.001)	0.000 (<0.001)	0.000 (0.005)
Eosinophils	0.001 (<0.001)	0.001 (<0.001)	0.005 (<0.001)	0.000 (0.174)
Basophils	0.038 (<0.001)	0.000 (0.137)	0.000 (<0.001)	0.000 (0.051)

#### 3.2.2 Age-stratified analysis

Age-stratified results are shown in **Table 2**. Basophil levels were significantly elevated post-pandemic across all age groups (*p* < 0.01), with an inverted U-shaped trend: the largest effect was in children aged 4–6 years (η² = 0.051), followed by 2–3 years (η² = 0.031) and 7–12 years (η² = 0.033); smaller effects were seen in ≤1 year (η² = 0.028) and 13–18 years (η² = 0.015). For all other CBC parameters, η² was ≤0.007 in every age subgroup, with no clear trend.

**Table 2:** Age-stratified group effects for CBC parameters (η², *p*).

Age group	WBC	Lymphocytes	Neutrophils	Eosinophils	Basophils
≤1 y	0.003 (0.370)	0.000 (0.852)	0.007 (0.192)	0.002 (0.462)	0.028 (0.008)
2–3 y	0.001 (<0.001)	0.000 (0.006)	0.001 (<0.001)	0.002 (<0.001)	0.031 (<0.001)
4–6 y	0.000 (0.006)	0.007 (<0.001)	0.001 (<0.001)	0.000 (0.012)	0.051 (<0.001)
7–12 y	0.001 (<0.001)	0.001 (<0.001)	0.001 (<0.001)	0.001 (<0.001)	0.033 (<0.001)
13–18 y	0.001 (0.012)	0.000 (0.521)	0.001 (0.008)	0.001 (0.126)	0.015 (<0.001)

### 3.3 Immunoglobulin E

#### 3.3.1 Whole-age model

In the whole-age GLM (**Table 3**), the pandemic group effect was significant (η² = 0.013, *p* < 0.001). Sex also showed a small effect (η² = 0.002), and the interaction was non-significant (*p* = 0.449). Age was not significant (*p* = 0.934).

**Table 3:** Whole-age general linear model results for IgE.

Source	Type III SS	df	Mean Square	F	Sig.	Partial η²
Corrected Model	7779243.411	4	1944810.853	23.955	<0.001	0.016
Intercept	79413337.876	1	79413337.876	978.177	<0.001	0.139
Age	559.418	1	559.418	0.007	0.934	0.000
Group	6424713.178	1	6424713.178	79.137	<0.001	0.013
Gender	1209381.805	1	1209381.805	14.897	<0.001	0.002
Gender×group	46532.769	1	46532.769	0.573	0.449	0.000
*R² = 0.016						

#### 3.3.2 Age-stratified analysis

Age-stratified analysis (**Table 4**) revealed that the pandemic-related increase in IgE was significant only in aged 7–18 years (η² = 0.017–0.018, *p* < 0.01). No significant differences were observed in younger age groups (*p >*0.05).

**Table 4:** Age-stratified group effects for IgE (η², *p*).

Age group	η² (group)	*P* value
≤1 y	0.000	0.078
2–3 y	0.000	0.887
4–6 y	0.000	0.979
7–12 y	0.017	<0.01
13–18 y	0.018	<0.01

## 4 Discussion

This large-scale, age-stratified study using data from a single tertiary hospital during the first two weeks of December of the year 2017, 2018, 2023 and 2024 revealed modest but significant increases in basophils and IgE in children after the COVID-19 pandemic, with distinct age-dependent patterns. Basophils showed an inverted U-shaped trend, peaking in children aged 4–6 years, while IgE increased only in aged 7–18 years. No other CBC parameters exhibited meaningful changes.

### 4.1 Strengths of the study design

By restricting data collection to the first two weeks of December each year, we minimized seasonal confounding. Excluding 2019 to 2022 reduced COVID-19 period biases. Strict quality control (removing cases with missing age/sex, IgE out of linear range, and hematologic disorders) enhanced data reliability. The use of emergency CBC data captured acute health status, while inpatient IgE data reflected more stable allergic profiles. Baseline comparison showed no sex difference between cohorts, and we adjusted for age in all analyses to account for the observed age difference, thereby reducing selection bias.

### 4.2 The age stratified pattern of Th2-skewed immune shifts

#### 4.2.1 Preschool window: basophil peak and immune plasticity

Basophils are key effector cells in Th2 immunity (7). The strongest pandemic-associated increase occurred in children aged 4–6 years, which known as the “immune programming window.” During this period, the thymus is highly active in generating naïve T cells, and the T-cell receptor repertoire is shaped by environmental exposures (15–17). The inverted U-shaped trend—rising from infancy, peaking in preschool years, then declining—parallels the developmental trajectory of Th2-type responsiveness, which is most malleable during early childhood (22, 23). Reduced microbial exposure due to lockdowns may have exaggerated this natural Th2 bias, leading to higher basophil levels at the peak of immune plasticity. The biological plausibility of this finding is supported by the critical role of microbial exposure in shaping immune tolerance during preschool years.

#### 4.2.2 Adolescent window: IgE elevation and cumulative effects

In contrast, IgE elevation was detectable only in adolescents (7–18 years). This delayed humoral response aligns with the time required for B-cell class switching, memory formation, and plasma cell differentiation. The prolonged exposure to pandemic-related lifestyle changes (continued reduced microbial diversity, altered dietary habits, decreased outdoor activity) likely accumulated over years, manifesting as increased IgE in adolescence. Puberty-related hormonal changes—particularly estrogen and testosterone—are known to modulate IgE production (24–26). The cumulative nature of IgE sensitization and the second wave of immune system remodeling during adolescence may amplify the effects of earlier environmental perturbations. This pattern—cellular changes (basophils) in preschool years preceding humoral changes (IgE) in adolescence—provide indirect supporting the hygiene hypothesis in a real-world setting (26–28).

### 4.3 Effect sizes and clinical relevance

Although statistically significant, effect sizes were small (η² ≤ 0.051 for basophils, 0.013–0.018 for IgE). This indicates that the pandemic explained only a small fraction of the total variability in these markers; individual factors such as genetics, nutrition, and prior infections dominated (11, 13). From a public health perspective, even small population-level shifts can translate into meaningful increases in allergic disease burden when applied to millions of children (3, 4). However, routine CBC and IgE measurements are not suitable for individual risk prediction.

### 4.4 Importance of age stratification

Our results highlight that the pandemic’s impact on immune parameters is not uniform across childhood. The identification of a preschool peak for basophils and an adolescent peak for IgE underscores the need for fine-grained age stratification in pediatric immunology research (18, 19). These age-specific windows may guide targeted monitoring and preventive strategies, such as focusing allergy screening efforts on preschool children for early immune deviations and on adolescents for emerging allergic sensitization. This pattern raises the hypothesis of a developmental sequence, but longitudinal studies are needed to confirm true temporal ordering.

### 4.5 Public health implications

Our findings suggest several actionable insights: Preschool children (4–6 years) may be a priority group for monitoring early Th2-skewed immune changes, potentially reflecting pandemic-related microbial deprivation. Adolescents (7–18 years) should be targeted for IgE-mediated allergic risk surveillance, as this group showed the only significant IgE increase. Encouraging safe outdoor activities and natural microbial exposure in these age groups could help maintain balanced immune development. Routine CBC and IgE are not recommended as individual screening tools but may be used in large-scale surveillance to detect population-level shifts.

### 4.6 Limitations and future directions

Several limitations should be acknowledged. First and most importantly, our analysis is based on routine laboratory biomarkers (basophil counts and total IgE) and does not include any clinical outcomes of allergic disease, such as asthma, allergic rhinitis, eczema, food allergy, wheeze, specific sensitisation, or allergen-specific IgE. Therefore, although we observed modest pandemic-associated shifts in these markers, we cannot infer that these changes translate into actual allergic disease burden or new-onset atopy in the population. The clinical significance of the observed biomarker differences remains unknown, and any public health implications should be considered purely speculative until outcome-based studies are available. Second, the cross-sectional design prevents assessment of within-individual changes; longitudinal follow-up would be more informative. Third, unmeasured confounders such as household environment, nutrition, and prior infections may have influenced results. Fourth, CBC and IgE data were obtained from different clinical settings (emergency vs inpatient), which may introduce selection bias. However, we compared baseline characteristics and found no sex difference; we also adjusted for age in all models to mitigate this bias. Fifth, individual-level data on COVID-19 infection history and vaccination status were not available, which precludes assessment of the specific contributions of infection-related and vaccine-related effects to the observed immune marker shifts. Future studies should combine longitudinal follow-up with detailed immunological profiling and clinical outcomes to fully elucidate long-term effects.

## 5 Conclusions

The COVID-19 pandemic was associated with modest increases in basophils and IgE in children, with distinct age-dependent patterns: basophil effects peaked in preschool children (4–6 years) and followed an inverted U-shape, while IgE effects were confined to adolescents (7–18 years). These findings were compatible with the hygiene hypothesis and suggest a temporal sequence of Th2-skewed immune shifts across childhood—cellular changes preceding humoral changes. All effect sizes were small, indicating that routine hematological parameters and IgE are not sensitive markers for individual monitoring. Future research should link these immunological shifts to allergic outcomes.

## Data Availability

The original contributions presented in the study are included in the article/Supplementary Material. Further inquiries can be directed to the corresponding author.
